# Plasma High Mobility Group Box 1 (HMGB1), Osteopontin (OPN), and Hyaluronic Acid (HA) as Admissible Biomarkers for Endometriosis

**DOI:** 10.1038/s41598-019-45785-w

**Published:** 2019-06-25

**Authors:** Yunlei Cao, Xishi Liu, Sun-Wei Guo

**Affiliations:** 1 Shanghai OB/GYN Hospital, Fudan University, Shanghai, 200011 China; 2 Shanghai Key Laboratory of Female Reproductive Endocrine-Related Diseases, Fudan University, Shanghai, China

**Keywords:** Endocrine reproductive disorders, Mechanisms of disease

## Abstract

Identification of biomarkers for endometriosis is an unmet medical need that demands to be fulfilled. In this study, we first used a mouse model of endometriosis and evaluated the potential utility of select biomarkers based on serial observations. Since fibrosis is the end result of lesional development, we chose high mobility group box 1 (HMGB1), osteopontin (OPN), and hyaluronic acid (HA), all three of them have been well documented to be involved in endometriosis and fibrosis, as potential biomarkers. In addition, we performed immunohistochemistry analysis of HMGB1, OPN, and the receptors for HMGB1, such as toll-like receptor 4 (TLR4), nuclear factor κB (NF-κB), proliferating cell nuclear antigen (PCNA), interleukin-33 (IL-33), and receptor for advanced glycation endproducts (RAGE)–a pattern recognition receptor, with HMGB1 being its important ligand. We then evaluated the same set of putative markers in 30 women with ovarian endometriomas and 20 without endometriosis, and reevaluated the 3 plasma markers 3 months after the surgical removal of all visible endometriotic lesions. In mouse, the lesional staining levels of OPN, RAGE, and IL-33 were all significantly higher than that of normal endometrium, and increased progressively as lesions progressed. In contrast to HMGB1, TLR4, p-p65 and PCNA staining levels were decreased progressively. In humans, lesional staining levels of OPN correlated positively, while that of HMGB1 correlated negatively with the extent of fibrosis. All three plasma markers correlated positively with the extent of lesional fibrosis. Through this integrated approach, we identified plasma HMGB1, OPN and HA as promising admissible biomarkers for endometriosis.

## Introduction

Endometriosis is a benign gynecological disease featuring ectopic deposition of endometrial-like tissues outside of the uterine cavity. A common disease affecting 6–10% of women of reproductive age^1^, endometriosis is one of the major contributors to dysmenorrhea, infertility, and chronic pelvic pain, impacting negatively on the quality of life and productivity in affected women^2^. The socioeconomic burden of endometriosis on health care expenses also is heavy^3,4^.

The enormous negative impact of endometriosis on many aspects of women’s lives calls for a speedy diagnosis and proper management. Unfortunately, as of now there is no non-invasive diagnostic procedure for endometriosis. Currently, visualization of endometriotic lesions commonly by laparoscopy or laparotomy, followed by histologic confirmation, is considered to be the gold standard for diagnosing endometriosis^5^. Yet this practice has its own risks, costs, and limitations^6^. The invasive nature of this diagnostic procedure and its associated cost, in conjunction with the requirement of skills, training, and experience for the operating physicians, contributes, perhaps in no small amount, to the well documented diagnostic delay in endometriosis. It often takes at least 5–6 years from the time a patient first experienced symptoms of endometriosis until she receives a definite diagnosis^7–9^. A recent review of the published literature finds that even this “gold standard” is not without any problems^6^.

Over the last 30 years, numerous attempts have been made to identify peripheral biomarkers of endometriosis. Dishearteningly, not a single published biomarker can be clinically validated^10–12^. Such an abject failure, by necessity, calls for a careful re-evaluation of the traditional ways of identifying biomarkers for endometriosis.

Ideally, “An ideal biomarker is one which is specific to the disorder, can be detected early in the disease process, accessible from peripheral tissue (non-invasive), stable, reproducible, and associated with a known mechanism”^13^. However, given the vast heterogeneity in location, size, color, depth of infiltration, presence or absence of adhesion, and other comobidity, let alone a kaleidoscopic variation in symptomology and severity, specificity is difficult to attain. In addition, evidence accumulated in the last few years indicates that endometriotic lesions are wounds undergoing repeated tissue injury and repair (ReTIAR) and, as such, undergo progressive changes, leading ultimately to fibrosis^14–17^. This dynamic and progressive nature of the disease course challenges the view that endometriosis as just one single, monolithic entity, nothing short of immutable, that may account for a seemingly disconnect between the changes in peripheral biomarkers and lesional progression and adds to the difficulty in identifying a biomarker that is specific to endometriosis. Intuitively, a genuine peripheral biomarker for endometriosis should, somehow, reflect the dynamic changes in lesions.

To certain extent, the putative markers exhibiting dramatic changes after surgical removal of endometriotic lesions may satisfy the requirement for specificity. This re-evaluation is important since the putative biomarker should be genuinely lesion-specific, in the sense that when lesions are present its peripheral concentrations should deviate from that of women without endometriosis. But once all lesions are removed, their concentrations should change, probably to a level that is close to or within the normal range, since the source for the deviation has been removed^18^. However, markers that meet this requirement may still not be specific enough to separate women with endometriosis from without. Therefore, the enormous complexity that is camouflaged behind the simple definition of endometriosis is likely responsible for the debacle that has been experienced in biomarker identification in endometriosis^10,12,19^ and demands study designs that are radically different from past studies.

In this study, we used an integrated approach to identify biomarkers. We first used a mouse model of endometriosis and evaluated the potential utility of select biomarkers based on serial observations. Since fibrosis is the end result of lesional development^14–17^, we chose high mobility group box 1 (HMGB1), osteopontin (OPN), and hyaluronic acid (HA), all three of them have been well documented to be involved in endometriosis^20–24^ and fibrosis^25–30^. In addition, we performed immunohistochemistry analysis of HMGB1, OPN, and the receptors for HMGB1, such as toll-like receptor 4 (TLR4), nuclear factor κB (NF-κB), and receptor for advanced glycation endproducts (RAGE)–a receptor for advanced glycation end-products (AGEs)^31^ and also considered as a pattern recognition receptor, with HMGB1 being its important ligand^32,33^, proliferating cell nuclear antigen (PCNA), and interleukin-33 (IL-33). We then evaluated the same set of putative markers in human endometriosis, and reevaluated 3 months after the surgical removal of all visible endometriotic lesions. Through this integrated approach, we identified plasma HMGB1, OPN and HA as promising and admissible biomarkers for endometriosis.

## Results

### Increasing total lesion weight as endometriotic lesions progress undisturbed

**Figure 1:**
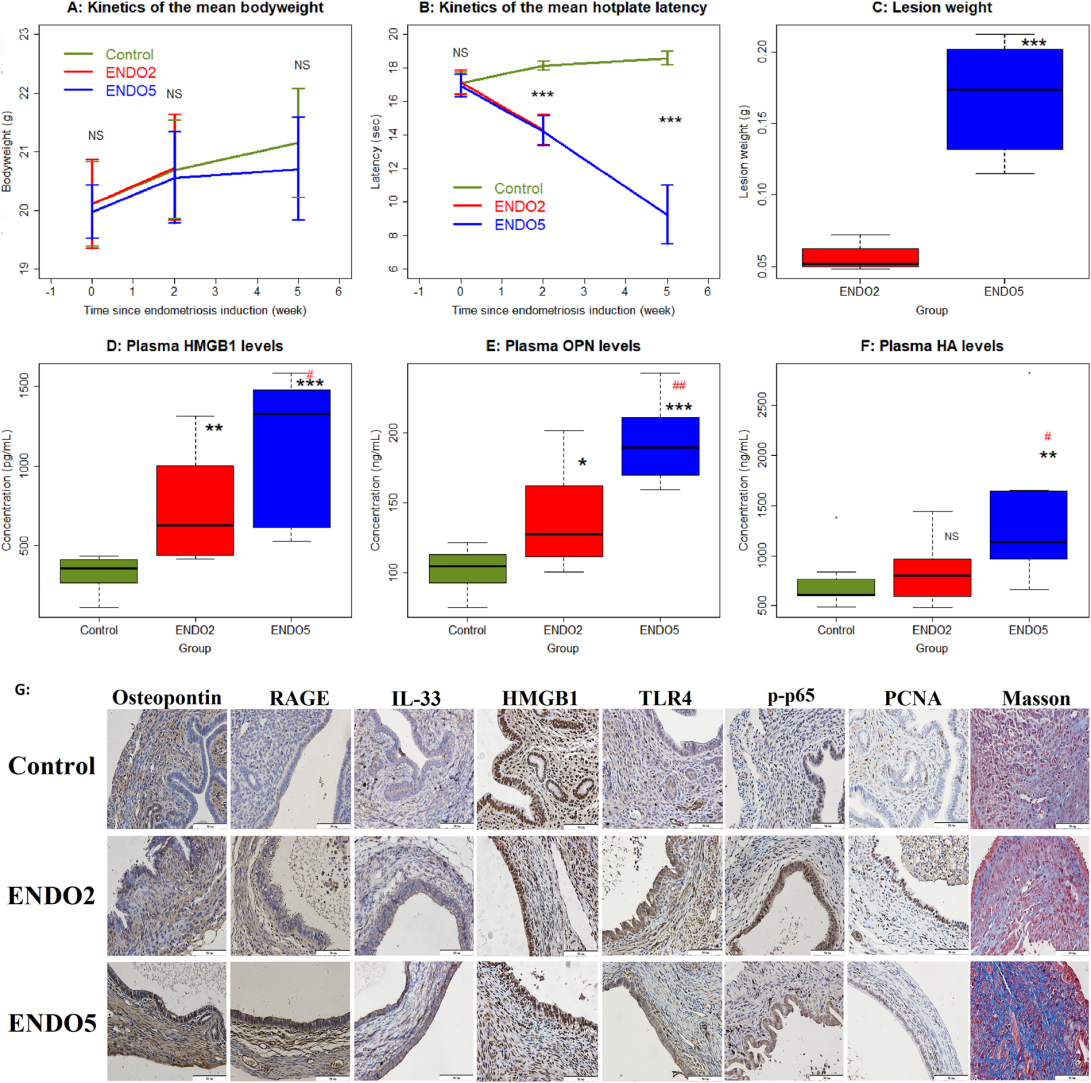
Summary results of the mouse experiment. (**A**) The dynamic changes of the mean bodyweight in different groups of mice. Data are presented as mean ± SD. (**B**) Kinetics of the mean hotplate latency in different groups. Data are presented as mean ± SD. (**C**) Boxplot of lesion weight in different groups. (**D**) Boxplot of the plasma HMGB1 levels (in pg/mL). (**E**) Boxplot of the plasma OPN levels (in ng/mL). (**F**) Boxplot of the plasma HA levels (in ng/mL). For all three groups, n = 8. Symbols of statistical significance levels: *or ^#^p < 0.05; **or ^##^p < 0.01; ***p < 0.001; NS: not statistically significant, i.e. p > 0.05, all by Wilcoxon’s rank test in reference to the control group (in black) or the ENDO2 group (in red) (except in (**A**,**B**), where the comparison was made by Kruskal’s test,). (**G**) Representative microphotographs of OPN, RAGE, IL-33, HMGB1, TLR4, p-p65, PCNA and Masson staining in CTRL mice and ENDO mice after 2 and 5 weeks of induced endometriosis. Bar = 50 µm.

No mouse died during the entire experimental course, and there was no significant difference in bodyweight among all three groups before and 2 weeks after the induction of endometriosis, and between mice from the control group (CTRL) and mice sacrificed 5 weeks after endometriosis induction (ENDO5) (all p-values > 0.26; Fig. 1A). As expected, there was no difference in hotplate latency among all 3 groups of mice before the induction (p = 0.80; Fig. 1B), but the difference became highly statistically significant 2 weeks after induction (p = 0.0005; Fig. 1B), and ENDO2 (sacrificed 2 weeks after endometriosis induction) and ENDO5 mice had statistically significantly shorter latency than that of the CTRL mice (both p-values = 0.0009). Indeed, the hotplate latency was significantly reduced 2 weeks after induction in both ENDO2 and ENDO5 mice (both p-values < 0.0002; Fig. 1B). The latency at week 5 in ENDO5 continued to deteriorate as compared with that at week 2 (p = 0.0078; Fig. 1B). Also as expected, the total lesion weight in ENDO5 mice was nearly 3 folds heavier than that of ENDO2 mice (167.5 ± 37.7 mg vs. 56.3 ± 8.7 mg, p = 0.00016; Fig. 1C). These results are consistent with what we reported previously^34^.

### Progression-dependent increase in plasma levels of HMGB1, OPN and HA with advancing lesions

We measured the plasma concentrations of HMGB1, OPN and HA in all mice and we found that the plasma levels of HMGB1, OPN and HA in ENDO5 mice were significantly higher than that of CTRL (all p-values < 0.007) and of ENDO2 mice (all p-values < 0.05; Fig. 1D–F). ENDO2 mice had significantly higher plasma HMGB1 and OPN, but not HA (p = 0.44), levels than that of the CTRL mice (p = 0.0019 and p = 0.021, respectively; Fig. 1D–F). Using the age of lesions (0 for CTRL mice) as an independent variable and regressing these putative plasma biomarkers suggested that all were associated with the lesional “age”, but the regression model for OPN had the highest *R*^2^ value (0.68, versus 0.52 and 0.33 for HMGB1 and HA, respectively; All models were statistically significant, as all p < 0.0033).

### Progression-dependent changes in immunostaining markers

We next evaluated the immunoreactivity against OPN, RAGE, IL-33, HMGB1, TLR4, p-p65, and PCNA, as well as the extent of fibrosis in endometriotic lesions. As shown in Fig. 1G, the immunoreactivity against OPN and IL-33 was seen in both epithelial and stromal components in normal endometrial and endometriotic tissues, and was localized in the cytoplasm, but the change was more prominent in epithelial cells. RAGE and TLR4 staining was seen mostly in cytoplasm and membranes in endometriotic, but not normal endometrial, epithelial cells. The immunoreactivity against HMGB1 staining was seen primarily in the nuclei in normal endometrial and endometriotic epithelial and stromal cells as well as cytoplasm in endometriotic epithelial cells. The p-p65 staining was seen in the nuclei as well as cytoplasm in endometriotic epithelial cells. The PCNA immunoreactivity was seen in endometriotic epithelial cells and some stromal cells and was localized in the nuclei, but the change in immunoreactivity as lesions progressed was more pronounced in epithelial cells.

**Figure 2:**
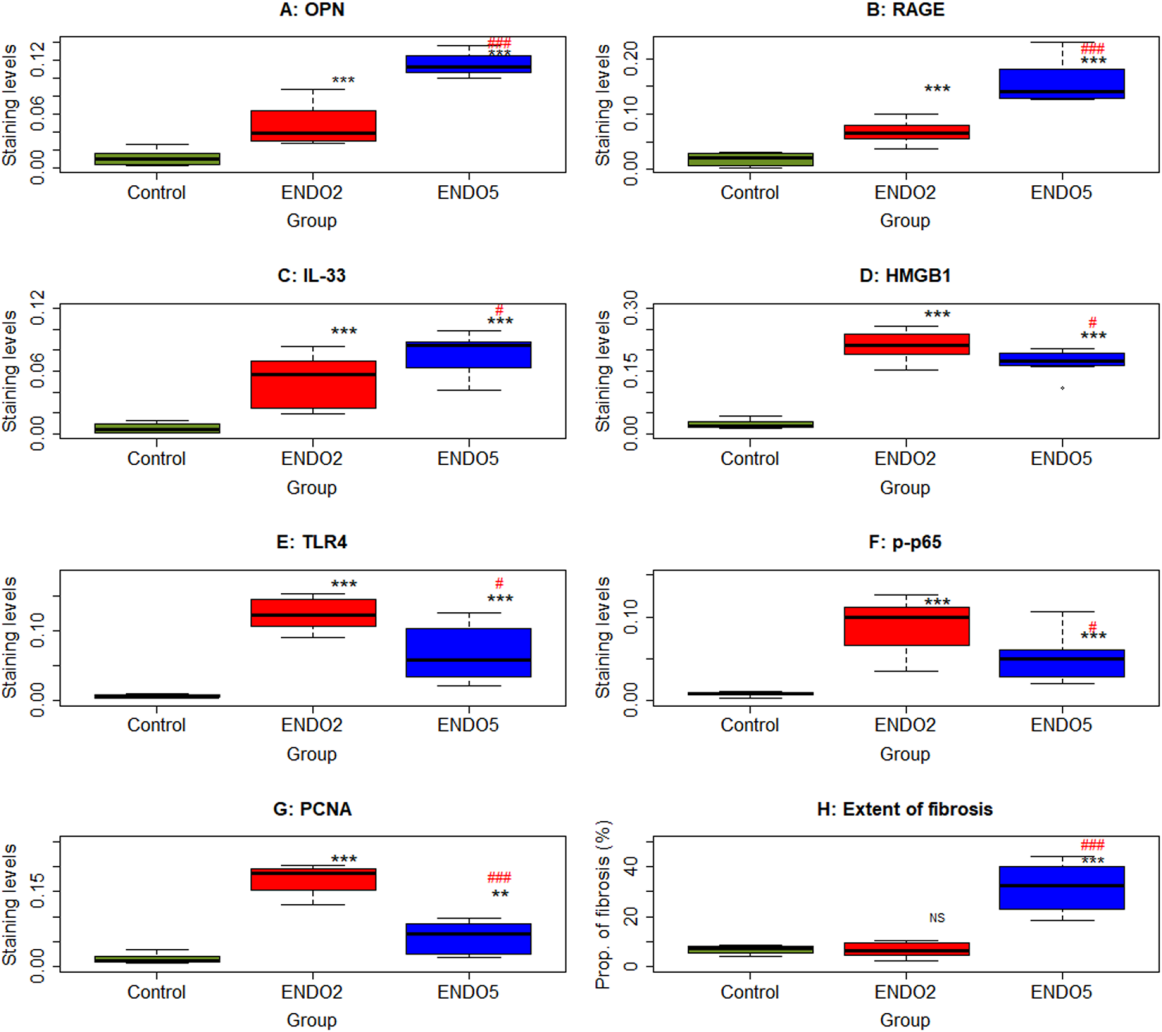
Summary immunostaining results of the mouse experiment. Boxplot of lesional OPN (**A**), RAGE (**B**), IL-33 (**C**), HMGB1 (**D**), TLR4 (**E**), p-p65 (**F**), PCNA (**G**) staining in the epithelial component, and the extent of lesional fibrosis (**G**) in different groups. For all three groups, n = 8. Symbols of statistical significance levels: *or ^#^p < 0.05; **or ^##^p < 0.01; ***p < 0.001; NS: not statistically significant, i.e. p > 0.05, all by Wilcoxon’s rank test in reference to the control group (in black) or the ENDO2 group (in red).

Compared to normal endometrium, the lesional staining levels of OPN, RAGE, and IL-33 were all significantly higher (all p-values < 0.00016; Fig. 2A–C). Interestingly, as lesions progressed, they were progressively increased (all 3 p-values < 0.038). The immunoreactivity against these 3 proteins was highly correlated (all r’s ranged from 0.85–0.94, and all p’s < 2.0 × 10^−7^).

Similarly, the HMGB1 staining levels in the cytoplasm of endometriotic epithelial cells in both ENDO2 and ENDO5 mice were also significantly elevated (both p = 0.00016; Fig. 2D), especially in ENDO2 mice. However, unlike OPN, RAGE and IL-33 staining, the HMGB1 staining levels were significantly reduced at lesions progressed (p = 0.028; Fig. 2D). Likewise, while the staining levels of TLR4 were all significantly higher in both ENDO2 and ENDO5 mice than that of CTRL mice (both p = 0.00016; Fig. 2E), they were significantly decreased as lesions grew older (p = 0.010). Consistently, similar pattern was seen in lesional p-p65 (p = 0.00016, p = 0.00016, and p = 0.028; Fig. 2F) and PCNA staining levels (p = 0.00016, p = 0.003, and p = 0.00016; Fig. 2G). The immunoreactivity against these 4 proteins was highly correlated (all r’s ranged from 0.85–0.97, and all p-values < 1.2 × 10^−7^).

Consistent with our previous report^34^, we found that the extent of lesional fibrosis in ENDO5, but not in ENDO2 (p = 0.96), mice was significantly elevated when compared to that of CTRL mice and was higher than that of ENDO2 mice (both p’s = 0.00016; Fig. 2H).

### Correlation with the lesional development stage

**Figure 3:**
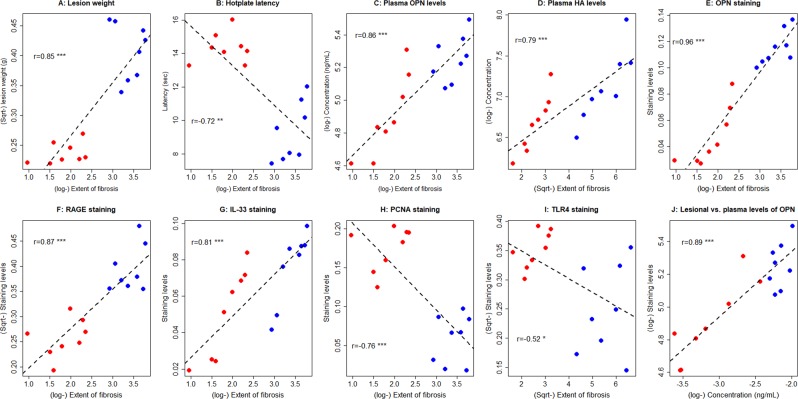
Relationship between the extent of lesional fibrosis and various factors. Scatter plot showing the relationship between the extent of lesional fibrosis and lesion weight (**A**), hotplate latency (**B**), plasma OPN levels (**C**), plasma HA levels (**D**), lesional staining levels of OPN (**E**), RAGE (**F**), IL-33 (**G**), PCNA (**H**), and TLR4 (**I**). (**J**) The scatter plot showing the relationship between the plasma OPN levels and lesional OPN staining levels. The dashed line represents the linear regression line, and the number is the Pearson’s correlation coefficient, followed by the symbols of statistical significance levels: *p < 0.05; **p < 0.01; ***p < 0.001.

Fibrosis appears to be the ultimate end result in lesions^15–17^. Therefore, we correlated the extent of lesional fibrosis with the immunostaining markers and the plasma markers we measured. The extent of lesional fibrosis correlated positively with the lesion weight (r = 0.85, p = 3.5 × 10^−5^; Fig. 3A), which essentially reflects the stage of lesional development in mice. It correlated negatively with the hotplate latency (r = −0.72, p = 0.0016; Fig. 3B), indicating the progressive nature of endometriosis. It also correlated positively with the plasma levels of OPN and HA (r = 0.86, p = 1.7 × 10^−5^ and r = 0.79, p = 0.0002, respectively; Fig. 3C,D), but not with that of HMGB1 (r = 0.41, p = 0.11). Within lesions, the extent of fibrosis correlated positively with the staining levels of OPN, RAGE, and IL-33 (all r’s ranged from 0.81–0.96, and all p-values < 1.5 × 10^−4^; Fig. 3E–G), but somewhat negatively with the PCNA and TLR4 staining levels (r = −0.76, p = 0.006, and r = −0.52, p = 0.04, respectively; Fig. 3H,I). The extent of fibrosis also seemed to be negatively correlated with lesional HMGB1 and p-p65 staining, but the correlation coefficients did not reach statistical significance (r = −0.43 and r = −0.35, both p’s > 0.092). Interestingly, while the plasma OPN levels correlated with the lesional OPN staining levels (r = 0.89, p = 3.6 × 10^−6^), the plasma HMGB1 levels did not correlate with the lesional HMGB1 staining (r = −0.32, p = 0.23).

The multiple linear regression analysis incorporating all 3 plasma biomarkers indicates that OPN was the only variable associated with the extent of lesional fibrosis (p = 1.7 × 10^−5^, *R*^2^ = 0.74). Within lesions, the extent of fibrosis was associated positively with OPN, RAGE, and IL-33 and negatively with HMGB1 (all p < 0.0009, *R*^2^ = 0.98), but not with p-p65, PCNA or TLR4 (all p > 0.25). These seem to suggest that OPN, RAGE, and IL-33 may be positively, while HMGB1, TLR4 and p-p65 may be negatively, associated with the progression of fibrosis.

In mice with endometriosis, the plasma OPN levels correlated positively with the lesional OPN staining levels (r = 0.89, p = 3.9 × 10^−6^; Fig. 3J). Interestingly, the plasma HMGB1 levels correlated negatively with the lesional staining levels, but it did not reach the statistical significance level (r = −0.32, p = 0.22).

Since the lesion weight represents a reasonable proxy for the development stage of lesions due to the homogeneity in all mice, we designated the lesion weight for the control mice as 0 and regressed the lesion weight with the 3 plasma markers. We found that all three putative markers were associated with the lesion weight, but the plasma OPN levels had the highest *R*^2^ value (0.71, versus 0.64 and 0.32 for HMGB1 and HA, respectively; all p < 0.005), suggesting that plasma OPN levels may be the best one among the three putative markers.

### Classification of mice based on putative plasma markers and hotplate latency

**Figure 4:**
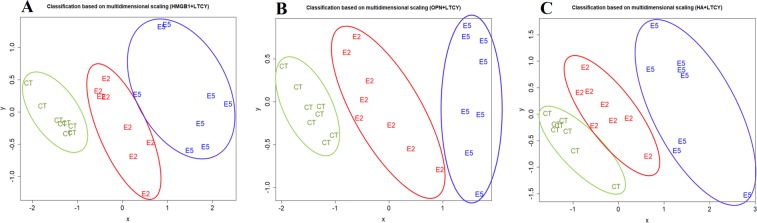
Results of multidimensional scaling analysis. Results of multidimensional scaling analysis using variables of hotplate latency (LTCY) and HMGB1 (**A**), OPN (**B**), and HA (**C**). Each character in the figure represents one data point from one mouse, with CT, E2, and E5 representing mouse from the control, ENDO2, and ENDO5 groups, respectively. Different groups of mice are all correctly classified.

From the above presentation, we can see that plasma HMGB1, OPN, and HA levels could be used as biomarker candidates for diagnosing endometriosis. To mimic the clinical situation, we grouped ENDO2 and ENDO5 mice as one group, i.e. mice with endometriosis, and used linear discriminant analysis to see which plasma marker has the potential as a biomarker. For the same reason, we also included the hotplate latency as a potential biomarker, since this variable could be a proxy for the severity of pain in women with endometriosis. Table 1 lists the sensitivity, specificity, sum of the two, and the correct classification rate for different models. Based on the criteria of parsimony and the performance, we can see from Table 1 that Model 5, i.e. the plasma HMGB1 level + hotplate latency, had an excellent performance, and the other models, such as Models 6 and 7, also had similar performance (Table 1). Indeed, each set of these 3 models could not only discriminate between mice with and without endometriosis, but also mice with different “ages” of lesions (Fig. 4). Thus, it can be concluded that plasma HMGB1, OPN, and HA levels are all biomarker candidates for endometriosis.

**Table 1:** Sensitivity, specificity, and correct classification rate for different models of linear discriminant analyses for the mouse experiment.

Model No.	Model	Sensitivity	Specificity	Sensitivity + specificity	Correct rate
1	HMGB1 alone	0.813	1.000	1.813	0.875
2	OPN alone	0.875	0.875	1.750	0.875
3	HA alone	1.000	0.000	1.000	0.667
4	Hotplate latency (LTCY) alone	0.938	1.000	1.938	0.958
**5**	**HMGB1** **+** **LTCY**	**1**.**000**	**1**.**000**	**2**.**000**	**1**.**000**
6	OPN + LTCY	0.938	1.000	1.938	0.958
7	HA + LTCY	0.938	1.000	1.938	0.958
8	HMGB1 + OPN + LTCY	1.000	1.000	2.000	1.000
9	HMGB1 + HA + LTCY	1.000	1.000	2.000	1.000
10	OPN + HA + LTCY	0.938	1.000	1.938	0.958
11	HMGB1 + OPN + HA + LTCY	1.000	1.000	2.000	1.000

Abbreviations used: LTCY: hotplate latency.

### Confirmation in human endometriosis

To confirm the results we found in mouse, we measured plasma levels of HMGB1, OPN, and HA in 30 patients with ovarian endometriomas (OE) and 20 women without endometriosis. The characteristics of recruited patients and controls are listed in Table 2.

**Table 2:** Characteristics of the recruited patients and controls.

Variable	Control (n = 20)	Endometriosis (n = 30)	*P* value
**Age (years)**			
*mean* ± *SD*	32.5 ± 6.1	32.2 ± 6.4	0.88
*Median* (*Range*)	31.5 (24–44)	31.5 (22–46)	
**Gravidity**			
*0*	6 (30.0%)	10 (33.3%)	0.68
*1*	7 (35.0%)	10 (33.3%)	
≥*2*	7 (35.0%)	10 (33.3%)	
**Parity**			
*0*	8 (40.0%)	13 (43.3%)	0.62
*1*	10 (50.0%)	16 (53.3%)	
≥*2*	2 (10.0%)	1 (3.3%)	
**Menstrual phase**			
*Proliferative*	13 (65.0%)	15(50.0%)	0.39
*Secretory*	7 (35.0%)	15 (50.0%)	
**Severity of dysmenorrhea**			
*None*	20 (100.0%)	10 (33.3%)	0.000
*Mild*		11 (36.7%)	
*Moderate*		5 (16.7%)	
*Severe*		4 (13.3%)	
**rASRM stage**			
*I*	NA	3 (10.0%)	NA
*Π*		5 (16.7%)	
*Ш*		10 (33.3%)	
*IV*		12 (40.0%)	
**rASRM score**			
*mean* ± *SD*	NA	39.3 ± 26.7	NA
*Median* (*Range*)		32.5 (4–92)	
**Other diseases**			
*No other diseases*	11 (55.0%)	16 (53.3%)	NA
*Adenomyosis*	—	—	
*Teratoma*	3 (15.0%)	—	
*Endometrial polyp*	3 (15.0%)	—	
*Physiologic cyst*	1 (5.0%)	—	
*paraovarian cyst*	—	4 (13.3%)	
*Adhesion*	—	7 (23.3%)	
*Corpus luteal cyst*	—	1 (3.3%)	
*DE*	—	2 (6.7%)	
*Recurrent abortion*	1(5.0%)	—	
*Vulval cyst*	1 (5.0%)	—	

Among the 30 patients, the rASRM scores were not correlated with the severity of dysmenorrhea (p = 0.75, Jonckheere-Terpstra trend test), nor the maximum or combined size of the endometriomas (both p’s > 0.30, Jonckheere-Terpstra trend test).

**Figure 5:**
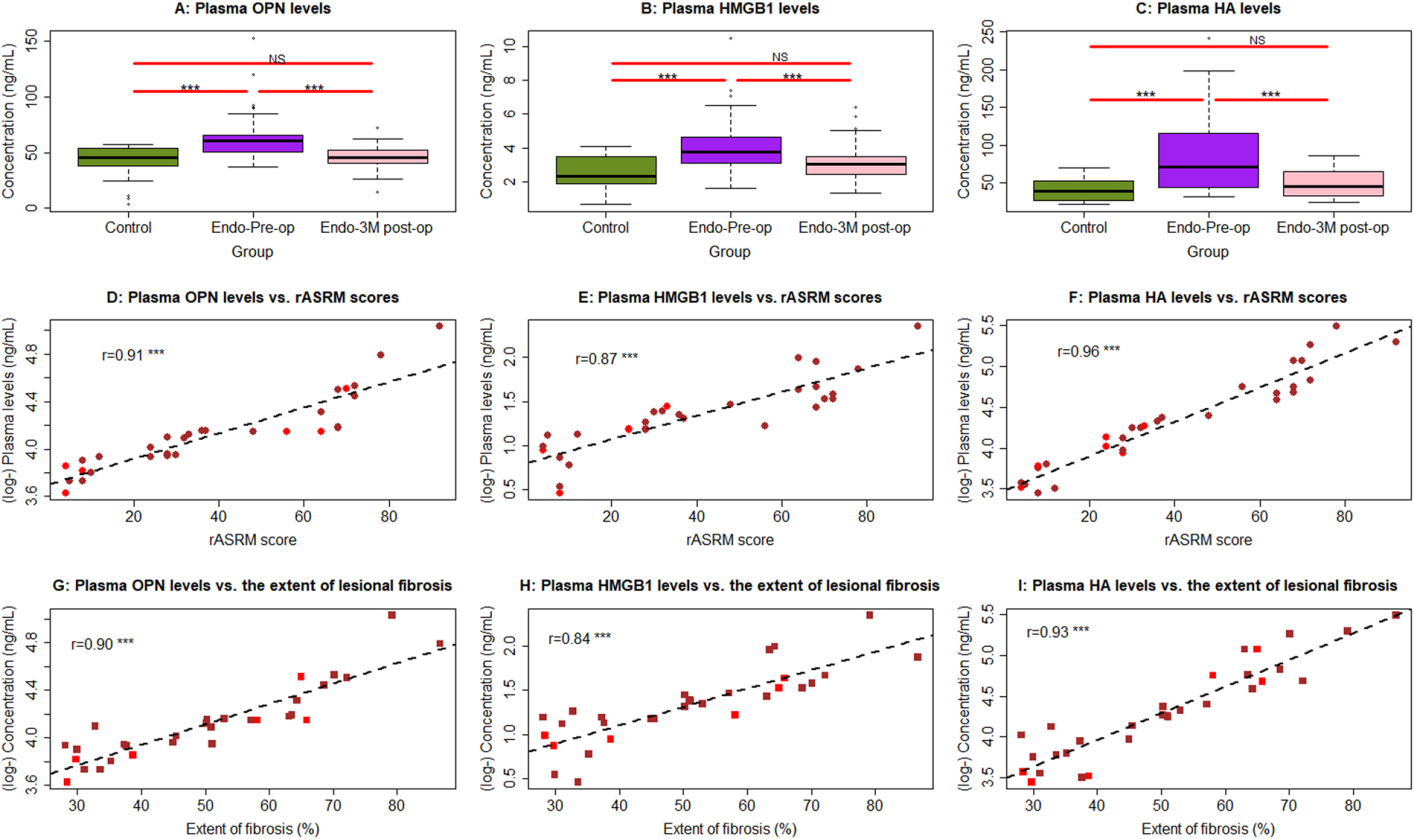
Summary of human data. Data summary of the plasma HMGB1 (**A**), OPN (**B**), and HA (**C**) levels in the peripheral blood from women without endometriosis (Control), women with ovarian endometriomas before the lesional-removal surgery (Endo-pre-op) and 3 months after the surgery (Endo-3M post-op). The statistical significance levels of the between-group difference in the designated groups are shown. Scatter plot showing the relationship between the rASRM scores and plasma OPN (**D**), HMGB1 (**E**), and HA (**F**). Scatter plot showing the relationship between the extent of lesional fibrosis and plasma OPN (**G**), HMGB1 (**H**), and HA (**I**). In (**D**–**H**), The dashed line represents the linear regression line, and the number is the Pearson’s correlation coefficient, followed by the symbols of statistical significance levels. Each dot in the figure represents one data point/patient, with the red and brown dots indicating the patient had unilateral and bilaterial ovarian endometriomas. Levels of statistical significance are ***p < 0.001; NS, nonsignificant, i.e. p > 0.05.

The preoperation plasma HMGB1, OPN and HA levels in women with endometriosis were significantly elevated when compared with that in controls (all p ≤ 0.0012; Fig. 5A–C). Three months after surgery–a period long enough for full recovery, however, their postoperative plasma levels were significantly reduced when compared with the preoperative level (all p ≤ 3.4 × 10^−5^) and apparently returned to the normal levels (all p ≥ 0.13; Fig. 5A–C). Multiple linear regression analyses incorporating age, parity, menstrual phase, co-occurrence with deep endometriosis or not, and group identity (endometriosis or control) indicated that for all three markers endometriosis was associated with higher preoperative plasma levels of these markers (all p < 0.0007, *R*^2^ ranged from 0.22 to 0.38). For HA, older age was also associated with higher levels (p = 0.014). The three markers positively correlated with the rASRM scores of endometriosis (r’s ranged from 0.87—0.96, all p < 4.4 × 10^−10^; Fig. 5D–F). However, none of the 3 markers was associated with the severity of dysmenorrhea in patients with endometriosis (all p > 0.35, Jonckheere-Terpstra trend test). In addition, while plasma levels of HMGB1 and OPN correlated with the cyst sizes and total volumes, that of HA did not (Supplementary Fig. S1).

We also performed IHC analysis of HMGB1, TLR4, RAGE, p-p65, PCNA, IL-33 and OPN—the same panel as that of the mouse experiment–in endometriotic lesions as well as Masson trichrome staining of endometriotic lesions. Since fibrosis is the terminal stage of endometriotic lesions, we divided the 30 patients with OE as moderately and highly fibrotic groups, with the former group comprising those whose lesions showed the proportion of fibrosis/collages less than 50% and the latter, no less than 50%.

**Figure 6:**
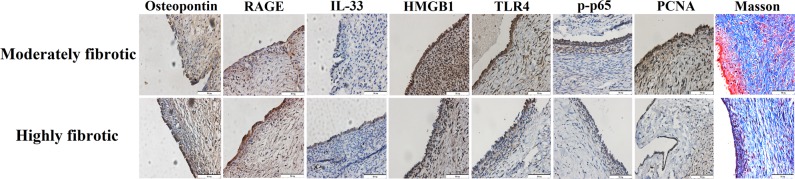
Representative microphotographs of immunohistochemical staining by the extent of lesional fibrosis. Representative microphotographs of lesional staining of OPN, RAGE, IL-33, HMGB1, TLR4, p-p65, and PCNA, and Masson staining from patients with endometriosis. Bar = 50 µm.

As shown in Fig. 6, the immunoreactivity against HMGB1 staining was seen in the nuclei in endometriotic epithelial and stromal cells as well as cytoplasm in endometriotic epithelial cells. TLR4 and RAGE staining was seen mostly in cytoplasm and membranes in endometriotic epithelial cells. The p-p65 staining was seen in nuclei as well as cytoplasm in endometriotic epithelial cells. PCNA immunoreactivity was seen in endometriotic epithelial cells and some stromal cells and was localized in the nuclei, but the change of immunoreactivity in epithelial cells was more prominent. For IL-33 and OPN, the staining was seen in both endometriotic epithelial cells and stromal cells, and was localized in the cytoplasm, but the change of immunoreactivity in epithelial cells was more prominent. We can see that moderately fibrotic lesions typically exhibited lower staining levels of OPN, RAGE and IL-33 but higher staining levels of HMGB1, TLR4, p-p65, and PCNA while highly fibrotic ones displayed higher staining levels of OPN, RAGE and IL-33 but lower staining levels of HMGB1, TLR4, p-p65, and PCNA. Overall, the results were consistent with those in the mouse experiment.

All three plasma markers correlated positively with the extent of lesional fibrosis (r’s ranged from 0.84—0.93, all p < 6.2 × 10^−9^; Fig. 5G–I). However, consistent with the mouse experiment which show no correlation between plasma HMGB1 levels and the extent of lesional fibrosis, such a correlation coefficient was the lowest among the three (Fig. 5G–I).

The rASRM scores correlated with the extent of lesional fibrosis (r = 0.95, p = 2.6 × 10^−15^). Using a Cox regression model incorporating co-variables such as age, parity, rASRM scores, total cyst volumes, and the immunostaining levels of HMGB1, TLR4, RAGE, p-p65, PCNA, IL-33 and OPN, we found that the severity of dysmenorrhea in women with endometriosis was significantly associated with the extent of lesional fibrosis (p = 0.019) and lesional HMGB1 staining levels (p = 0.026).

**Figure 7:**
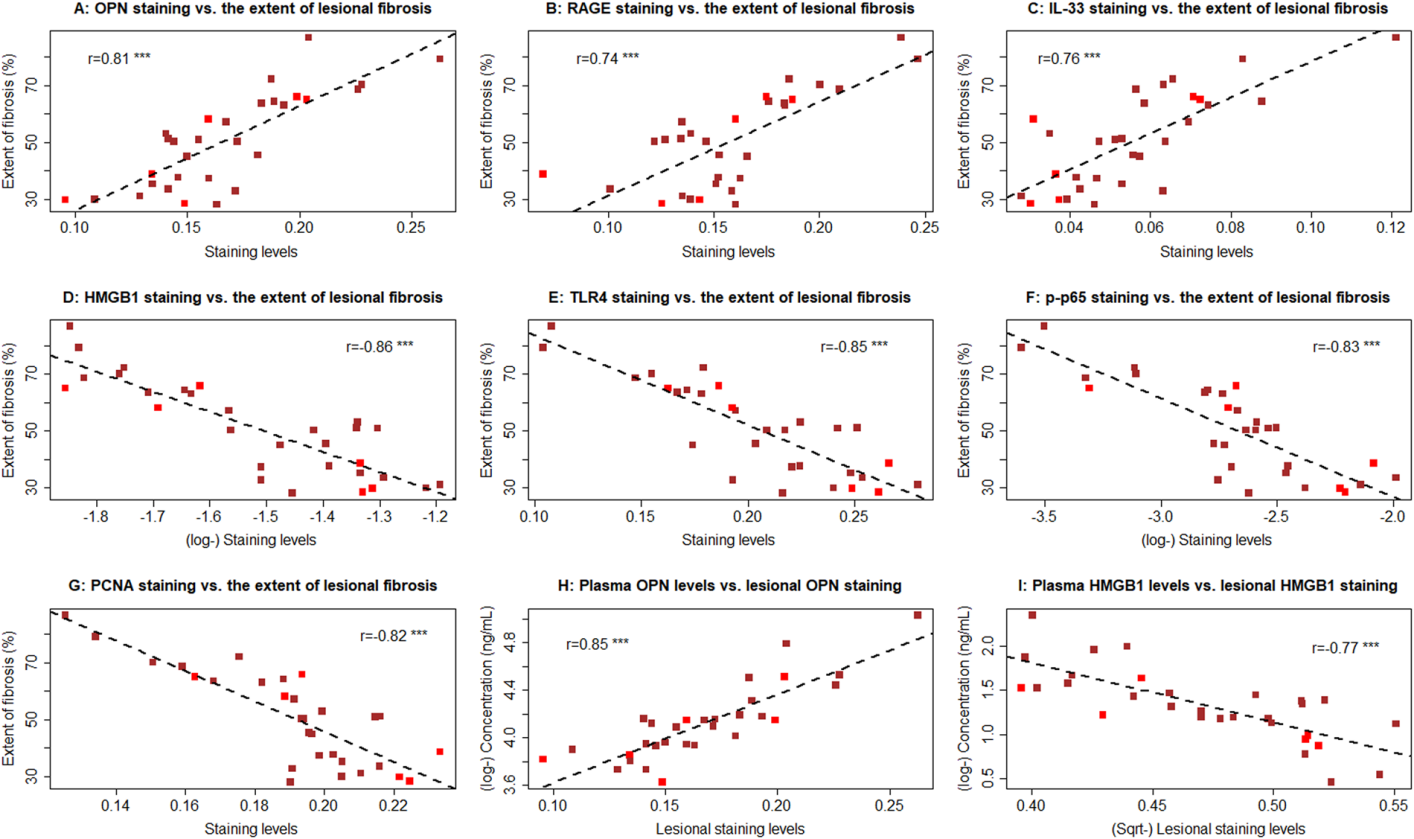
Relationship between the extent of lesional fibrosis and various factors. Scatter plot showing the relationship between the extent of lesional fibrosis and lesional staining of OPN (**A**), RAGE (**B**), IL-33 (**C**), HMGB1 (**D**), TLR4 (**E**), p-p65 (**F**), and PCNA (**G**) in the epithelial component, and plasma levels of OPN (**H**) and HMGB1 (**I**). The dashed line represents the linear regression line, and the number is the Pearson’s correlation coefficient, followed by the symbols of statistical significance levels: ***p < 0.001. Each dot in the figure represents one data point/patient, with the red and brown dots indicating the patient had unilateral and bilateral ovarian endometriomas.

Within endometriotic lesions, the extent of fibrosis correlated positively with the staining levels of OPN, RAGE, and IL-33 (r’s ranged from 0.74—0.81, all p-values < 3.2 × 10^−6^; Fig. 7A–C) but negatively with that of HMGB1, TLR4, p-p65 and PCNA (r’s ranged from −0.86— −0.82, all p-values < 3.5 × 10^−8^; Fig. 7D–G). Interestingly, the plasma OPN levels correlated positively with the lesional staining levels (r = 0.85, p = 2.8 × 10^−9^; Fig. 7H), but the plasma HMGB1 levels correlated negative with the lesional staining levels (r = −0.77, p = 6.7 × 10^−7^; Fig. 7I).

Overall, there were essentially two groups of lesional markers, one consisting of OPN, RAGE and IL-33, and the other, HMGB1, TLR4, p-p65 and PCNA. Within the first group, the staining levels of any two members were highly correlated (r’s ranged from 0.69 to 0.80, all p < 2.6 × 10^−5^), and the same was true for the second group (r’s ranged from 0.86 to 0.94, all p < 1.6 × 10^−9^). Each member in the first group was negatively correlated with each and every member from the second group. The extent of lesional fibrosis correlated positively with any member in the first group but negatively with that of the second group.

### Classification based on putative plasma markers and dysmenorrhea severity

**Figure 8:**
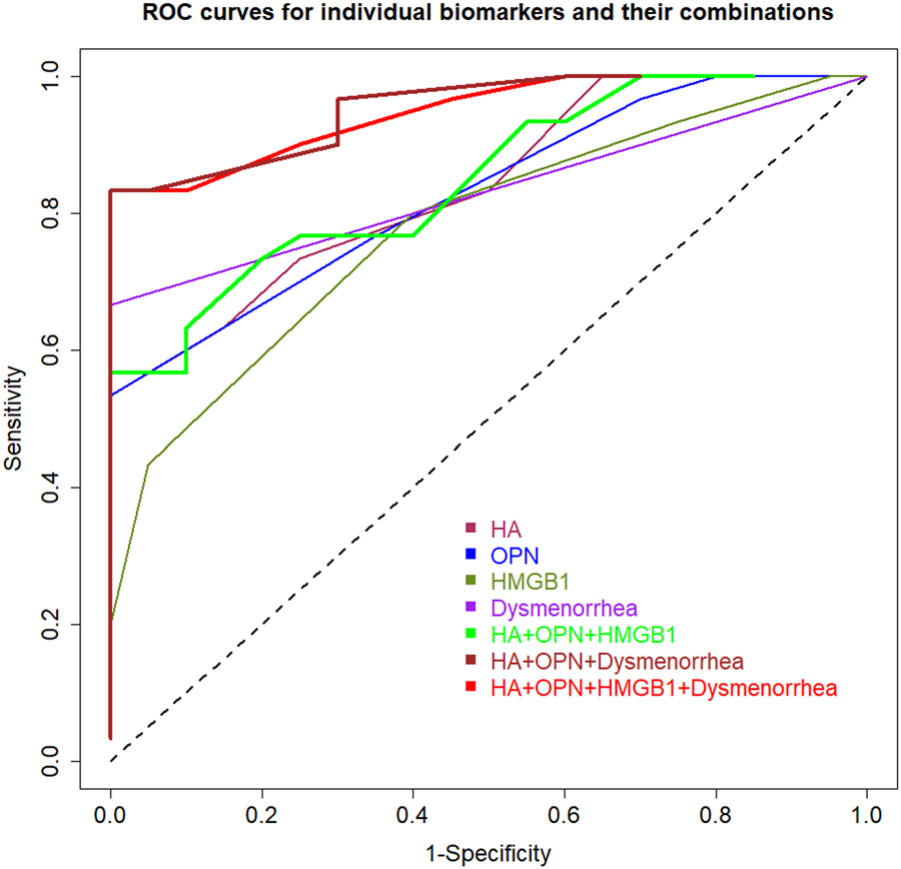
Receiver operating characteristic (ROC) curves for individual markers and their select combinations. Different ROC curves are represented by curves of different colors. The dashed diagonal line represents the ROC curve under a random classifier.

We performed linear discriminant analyses using different combinations of plasma biomarkers and the severity of dysmenorrhea to see their performance. Table 3 lists the sensitivity, specificity, sum of the two, the correct classification rate and the area under the curve (AUC) for different models. The receiver operating characteristic (ROC) curves for individual markers and their select combinations are presented in Fig. 8. We can see that overall the performance of the combination of the 3 plasma markers is better than that when used alone (Fig. 8). Severity of dysmenorrhea seemed to perform quite well, but it is likely due to the absence of any women with dysmenorrhea in the control sample. Also, despite the fact that Model 14 has one less marker (plasma HMGB1) than Model 15, its performance is no worse (Fig. 8).

**Table 3:** Sensitivity, specificity, correct classification rate and AUC values for different models of linear discriminant analyses for human data.

Model No.	Model	Sensitivity	Specificity	Sensitivity + specificity	Correct rate	AUC
1	HMGB1 alone	0.800	0.600	1.400	0.720	0.778
2	OPN alone	0.867	0.450	1.317	0.700	0.829
3	HA alone	0.733	0.750	1.483	0.740	0.839
4	Severity of dysmenorrhea (DYS)	0.667	1.000	1.667	0.800	0.833
5	HMGB1 + OPN	0.767	0.600	1.367	0.700	0.846
6	HMGB1 + HA	0.733	0.650	1.383	0.700	0.823
7	OPN + HA	0.767	0.650	1.417	0.720	0.849
8	HMGB1 + DSY	0.733	1.000	1.733	0.840	0.898
9	OPN + DSY	0.767	1.000	1.767	0.860	0.943
10	HA + DSY	0.767	1.000	1.767	0.860	0.917
11	HMGB1 + OPN + HA	0.733	0.750	1.483	0.740	0.848
12	HMGB1 + OPN + DSY	0.767	1.000	1.767	0.860	0.946
13	HMGB1 + HA + DSY	0.800	1.000	1.800	0.880	0.927
14	**OPN** + **HA** + **DSY**	**0**.**833**	**1**.**000**	**1**.**833**	**0**.**900**	**0**.**953**
15	HMGB1 + OPN + HA + DSY	0.800	1.000	1.800	0.880	0.948

Abbreviations used: DYS: severity of dysmenorrheal; AUC: area under the curve.

**Figure 9:**
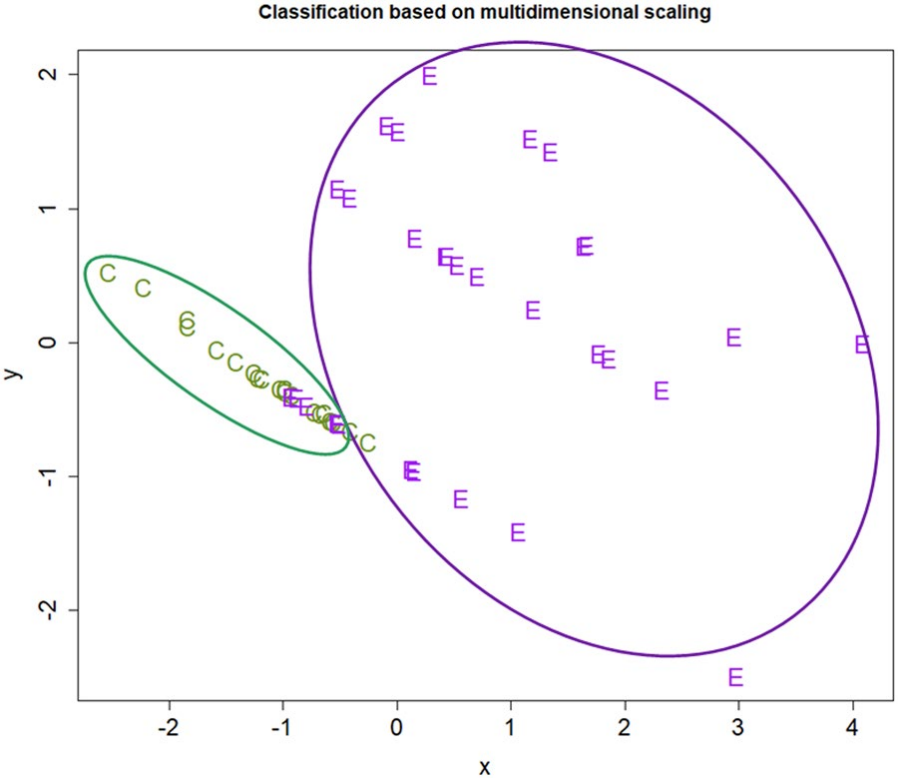
Results of multidimensional scaling analysis of human data. Results of multidimensional scaling analysis using variables of the severity of dysmenorrhea (DYS), OPN, and HA. Each character in the figure represents one data point from one patient, with E and C representing patient with endometriosis and without, respectively.

Based on the criteria of parsimony and the performance, we can see from Table 3 that Model 14, i.e. the plasma OPN and HA levels + severity of dysmenorrhea, had the best performance. Of course, other models may also be equally admissible. Based on multidimensional scaling, Model 14 discriminated between patients with endometriosis and without fairly nicely, although there were still some misclassification (Fig. 9). Taken together, it can be concluded that plasma HMGB1, OPN, and HA levels are all admissible biomarkers for endometriosis.

## Discussion

In this study, using a radically different study design—theory-based, targeted discovery from animal experimentation based on the knowledge of the natural history of endometriotic lesions and then validation from human samples, we found that plasma HMGB1, OPN, and HA are promising admissible biomarkers for diagnosing endometriosis.

HMGB1, OPN, and HA have all been reported to be involved in wound healing^35–39^ and their plasma concentrations have been reported as potential biomarkers of fibrosis^25–30^. It is well documented that tissue injury triggers evolutionarily conserved mechanism of tissue repair, manifested as a well-orchestrated repairing phases of hemostasis, inflammation, fibroblast proliferation, myofibroblast differentiation, and deposition of extracellular matrix (ECM)^40^. In particular, damaged cells release intracellular molecules, called damage-associated molecular patterns (DAMPs), that activate innate immunity just like pathogen-associated molecular patterns (PAMPs)^41^. PAMPs and DAMPs activate identical pattern recognition receptors including toll-like receptors (TLRs) and inflammasomes, which have already been implicated in endometriosis^20,42,43^. TLRs activation can also induce NF-κB dependent production of inflammatory cytokines and chemokines^44^, which have also been implicated in endometriosis^45,46^.

Similar to HMGB1, IL-33 also is an alarmin due to its participation in tissue homeostasis^47^. Through the IL-1 receptor-related suppression of tumorigenicity 2 receptor (ST2), IL-33 acts on immune cells associated with type 2 and regulatory immune responses, including type 2 innate lymphoid cells (ILC2s), Th2 cells, myeloid-derived suppressor cells, and Tregs^47^ and induces alternatively activated macrophages^48,49^. Not surprisingly, IL-33 as an alarmin has been implicated in endometriosis^50^.

However, if the wound-healing responses during tissue repair becomes unbridled, it leads to pathological conditions. In contrast to normal wound healing, a persistent accumulation of myofibroblasts, which are the major effector cells that synthesize a variety of ECM proteins and HA in the fibrotic tissue^51,52^, in particular, is associated with the pathological reorganization and expansion of ECM components, resulting in fibrosis^53^. The deposition of HA in the pericellular matrix signals one of the earliest fibrogenic responses^54^.

HA is a nonsulfated glycosaminoglycan produced by mesenchymal cells and many tumor cells and its action is mediated through its cell surface receptor CD44^55,56^, which plays an important role in inflammatory cell recruitment^57,58^ and activation^59,60^. Accumulation of HA is a characteristic of disorders that are associated with progressive tissue fibrosis^61^. In particular, increased production of HA due to overexpression of HA synthase 2 (HAS2) by myofibroblasts is reported to be responsible for severe lung fibrosis^52^.

OPN is a multifunctional matricellular glycoprotein produced by a broad range of cells, including osteoclasts, immune cells and fibroblasts^62–64^. It binds to cell surface integrins and CD44 within the ECM and modulates signaling in a wide variety of cell types^63,64^. OPN is required in TGF-β1-induced fibroblast-to-myofibroblast transdifferentiation (FMT)^65^.

RAGE may stimulate proliferation of fibroblasts^66^. The likely elevated AGEs^67^ due to increased oxidative stress^68^ may also induce RAGE and facilitate lesional fibrogenesis through TGF-β1^69^ or angiotensin I pathways^70^.

Given overwhelming evidence that endometriotic lesions are wounds undergoing ReTIAR and subsequent fibrogenesis^14–17,71^, the choice of plasma HMGB1, OPN, and HA is biologically justifiable. Our human data are consistent with the mouse data, and are consistent with a previous report that OPN levels are elevated in women with endometriosis^22^ and with the report that TLR4 expression dose-dependently increases with the HMGB1 stimulation^20^.

OE can be efficiently identified by ultrasound, which is now widely available and is inexpensive^72^. Since this study used patients with OE in human study, naturally there is a question as whether the biomarkers identified through the integrated approach is superfluous. However, even in the hands of most experienced sonologists, there is still a sizable portion of patients, about 10%, cannot be accurately diagnosed^72,73^. That is, there is still ample room for improvement when it comes to diagnosing endometriosis, and thus the need for biomarkers. In addition, the rationale of choosing the 3 particular markers in this study is that these markers may reflect the extent of lesional fibrosis, a proxy for lesional development stage. There is growing evidence to show different subtypes of endometriosis, such as OE, deep endometriosis, and perhaps peritoneal endometriosis as well, have the same natural history, in the sense that they all go through the same molecular and cellular processes, i.e. epithelial-mesenchymal transition (EMT), FMT, smooth muscle metaplasia (SMM), and fibrogenesis but they differ in the extent and/or thoroughness and completeness^74^, which, in turn, can be explained by their different microenvironments^75,76^. In other words, while different subtypes of endometriosis may have different pathogenesis, their pathophysiology is very much alike. Thus, their lesions (including adenomyotic lesions as well^77,78^) have similar, if not identical, natural history^14^. Importantly, to serve as biomarkers, it would be ideal if their variation can also reflect the developmental stage of the underlying endometriotic lesions. It is not necessary to require that the biomarkers have to reflect as how the lesions got started. Thus, our choice of the 3 markers is not for OE *per se*. And this can be easily seen from the results of our mouse experiment, which was just induced peritoneal endometriosis, not OE. Nonetheless, the results from the mouse experiment are very similar to what we found in OE, supporting our argument. Of course, further studies in women with deep/peritoneal endometriosis are needed to verify this.

Our results are in broad agreement with previously reported overexpression of RAGE^21,79^, TLR4^42^, NF-κB p-p65^80,81^, OPN^23,82^ in endometriotic lesions. Our study also is consistent with the reported higher CD44 expression in endometriosis^83,84^, and the report that inhibition of HA synthesis hinders angiogenesis in developing endometriosis^24^, which implicate the involvement of HA. The increased serum and peritoneal IL-33 levels in women with endometriosis^85,86^ also agree with our findings.

However, our mouse study also indicates that, as endometriotic lesions progress, their HMGB1, TLR4, p-p65 and PCNA expression decreases, although their expression levels are still higher than that of normal endometrium. This likely due to the fact that, while early wound healing is mostly characterized with inflammation, the later stage is characterized more predominantly with tissue repairing^40^. Alternatively, IL-33 has been shown to bind the p65 subunit of NF-κB and inhibit NF-κB transcriptional activity^87^, which may account for reduced expression of TLR4, HMGB1 and PCNA.

As part of the biomarker panel, the demographic, reproductive and clinical information such as age, parity, presence or absence of different symptoms and their severity, have been shown to be important in patients with endometriosis and, as such, need to be carefully collected and taken into consideration^6^. In addition, due to the changing nature of endometriotic lesions, the biomarker panel should ideally profile the suspected patients from multiple and different angles, requiring biomarkers that profile different aspects of the disease, such as level of coagulability^18^ and the stage of fibrogenesis. Since the potential patients are surely heterogeneous with respect to location, size, color, depth of infiltration, presence or absence of adhesion, comobidity, along with a kaleidoscopic variation in symptomology and severity as well as the progressive nature of the disease course, by necessity large samples or big data are required to account for most, if not all, possible combinations of these parameters in order to successfully identify patients with endometriosis from those without. This poses a formidable challenge in resources, time, and efforts to a single research team, and collaboration may be the only solution. But this would require the harmonization of sample collections and outcome measures^88,89^. In other words, there will be a long way to go before we can have a panel of biomarkers that are clinically useful in diagnosing endometriosis, especially in differential diagnosis. Until then, the biomarkers identified through a rigorous study can only be termed as *admissible* biomarkers, awaiting for further validation.

Our mouse and human studies both demonstrate that, as lesions grow older and become more fibrotic, the lesional staining of OPN, RAGE and IL-33 becomes more intense while that of HMGB1, TLR4 and PCNA is gradually reduced, even though their staining levels were all significantly higher than normal endometrium. This dynamic change in staining and possibly in gene expression as lesions progress serves as a reminder that an endometriotic lesion is not a static, monolithic, and certainly not an immutable, entity^14^. Rather, it will progressively become more fibrotic if undisturbed, concomitant with changes in gene transcription, protein translation, along with morphology, appearance and coloration. This view underscores the point that not only gene/protein expression levels but also the peripheral levels of certain markers would be different for patients with endometriotic lesions at different stages.

This study has several strengths. First, we capitalized our knowledge on the newly unveiled natural history of endometriotic lesions, and focused on peripheral markers that are potentially related with the extent of lesional fibrosis, which is known to be the end result of lesional development^14^. Second, we designed a mouse study that evaluated the potentials of the peripheral markers and their changes with the progression of lesions. This dynamic view gave us the advantage of viewing endometriosis not as a static entity, but, rather, a changing, dynamic, and progressive disease. This approach should maximize our chance of finding the right biomarker that will be clinically useful. Third, we re-evaluated the plasma markers 3 months after the surgical removal of all endometriotic lesions, and made the requirement that these markers should change as a result of lesional removal. Lastly, by evaluation of both peripheral markers and lesional staining markers simultaneously, we have not only gained a better understanding of the putative biomarkers and their relationship with endometriotic lesions but also exposed areas in need for further research.

This study also has its limitations. First, we did not use a validation sample to further evaluate the performance of the putative biomarkers. This is mainly due to the constraints of time and resources. As a result, the estimates of sensitivity, specificity and other performance parameters presented in this study are likely inflated since they were based on the training data only. The real performance parameters in independent samples are likely to be worse. Future studies are needed to reevaluate the model performance. Second, in our control group no women had dysmenorrhea while in women with OE various severity of dysmenorrhea was reported. It is known that women without endometriosis can still have dysmenorrhea^90^ even though the prevalence may be much lower than those with endometriosis. Our choice of the control women did not deliberately exclude those complained of dysmenorrhea, and the resultant difference is largely a chance event. However, due to the sharp difference in the severity of dysmenorrhea, the performance of the classifer incorporating the severity of dysmenorrhea was improved substantially, as can be seen from the ROC curves (Fig. 8). Thus, the sensitivity and specificity estimates based on its use are very likely to be inflated, especially when in real clinical situation when women with other gynecological disorders may also complain of dysmenorrhea. This calls for the inclusion of symptomology as a part of the biomarker-based classifier. Third, while we performed immunostaining for HMGB1 and OPN for endometriotic lesions, we did not evaluate HA staining, nor did we stain, perhaps more appropriately, hyaluronan synthases (HASs) and/or hyaluronidases, or CD44, the receptor of HA. Both HASs and CD44 have been implicated in the development of endometriosis^24,91,92^, but the role of hyaluronidases remains unclear. The lesional staining of HASs, hyaluronidases and CD44 would probably have provided us with more information regarding their relationship with the plasma levels of HA. Similarly, we did not evaluate lesional staining of ST2, the receptor for IL-33, which is very likely to be elevated. Future investigations are warranted to delineate the roles of CD44, HASs, hyaluronidases and ST2 in the development of endometriosis. Fourth, we did not include ultrasonic findings in our analysis. Ultrasonic examination can pretty much determines the location, size and content of the endometrioma lesions. Finding the associations, if any, between certain ultrasonic features of the endometriomas and the putative biomarkers should be very meaningful endeavor and should be pursued in future studies. Fifth, a panel of ideal biomarkers should be able to inform physicians as what subtype of endometriosis the patient has, but, since our study only evaluated patients with mostly OE, we were not be able to do this analysis. Future research is justified to look into this important issue. Lastly, our sample sizes are too modest to include different subtypes of endometriosis with varying symptomology, stage, co-morbidity, age and other potential confounding factors. The sample size for the control group also is very moderate and did not include women with dysmenorrhea or gynecological conditions other than endometriosis and/or adenomyosis. As such, extreme caution should be exercised when one wishes to extrapolate our findings to more general settings and/or differential diagnosis. Future studies with much sample sizes are warranted.

In summary, plasma HMGB1, OPN, and HA are promising admissible biomarkers for diagnosing endometriosis. However, future studies are warranted to validate their performance in real clinical settings.

## Materials and Methods

### Reagents

Anti-human/mouse antibodies against TLR4, RAGE, NF-κB phosphorylated p65 subunit (p-p65), PCNA, OPN and IL-33 were purchased from Abcam (Cambridge, MA, USA), and anti-human/mouse antibody against HMGB1 from Proteintech Group, Inc. (Chicago, IL, USA). Human-specific and mouse-specific HMGB1 ELISA Kits were purchased from MyBioSource (San Diego, CA, USA), and human- and mouse-specific HA and OPN (specific to both humans and mouse) ELISA Kits were purchased from R&D systems (Minneapolis, MN, USA).

### Mice and the induction of endometriosis

Thirty-two female 6–8 weeks old Balb/c mice, 18–20 g in bodyweight, were purchased from Shanghai Slack Experimental Animals Co., LTD (SCXK-2007-0005, Shanghai, China) and used for this study. All mice were maintained under controlled conditions with a light/dark (12/12 h) cycle, and had free access to chows and water *ad libitum*. The animal experiment was performed under the guidelines of the *National Research Council’s Guide for the Care and Use of Laboratory Animals*^93^ and approved by the institutional experimental animals review board of Shanghai OB/GYN Hospital, Fudan University.

Among 32 mice, 8 mice were randomly selected as donors of uterine tissue fragments. They were initially treated i.m. with 100 mg/kg estradiol benzoate (Animal Medicine Factory, Hangzhou, China) twice a week after one week of acclimation. The remaining 24 mice were designated as recipients.

One week after the treatment of donor mice with estrogen, the recipient mice were divided into two groups, ENDO mice (n = 16) that received uterine tissue fragments from donor mice and CTRL mice (n = 8) that received normal saline only, all through intraperitoneal (i.p.). For ENDO mice, endometriosis was induced using the established induction model as first described in^94^ and used in our previous studies^95,96^. Briefly, after sacrifice the uteri tissues from donor mice were harvested in a Petri dish, washed with sterile saline twice and then split longitudinally. Finally, the uterine tissues were minced into fragments with their diameters small than 1 mm, then the uterine fragments were suspended in sterile saline and injected into the abdominal cavities of recipient mice. Each donor mouse’s uterus was injected in equal amount to two recipient mice. An equal volume of sterile saline was injected to control mice. The day when the induction procedure was performed was designated as Day 0.

Bodyweight and hotplate latency were measured for all mice at Day 0 prior to i.p. injection and then 2 and 5 weeks after induction (before sacrifice). At the end of the 2^nd^ week after induction, the ENDO mice were further divided, at random, into equal-sized two groups: ENDO2 and ENDO5. Mice in the ENDO2 group were sacrificed immediately, while those in the ENDO5 group were allowed to stay but were sacrificed 5 weeks after induction. The CTRL mice were sacrificed at the same time as those ENDO5 mice. Before sacrifice, a peripheral blood sample was collected in tubes with anticoagulant ethylenediaminetetraacetic acid (EDTA) from all mice, and the plasma samples were obtained by centrifuging the blood samples at 3000 rpm for 10 minutes at room temperature and stored at −80 °C immediately until further analyses. After sacrifice, the endometriotic lesions in ENDO mice were carefully excised, weighed and then processed for further analysis, and the uteri of CTRL mice were excised and then processed. Both lesional and uterine tissue samples were fixed in neutral-buffered formalin for histologic, immunohistochemical, and histochemical analyses.

### Patients and specimens

We also recruited 30 premenopausal patients with laparoscopically and histologically diagnosed OE. Among them, 7 were found to have peritoneal adhesion, 2 had deep endometriosis (DE), 4 had paraovarian cyst, 1 had corpus luteal cyst. All patients were scored following the rASRM classification during surgery. For controls, we recruited 20 age-matched, in frequency, women without endometriosis. Among them, 3 (15%) had teratoma, 3 (15%) with endometrial polyp, 1 (5%) each had physiologic cyst, recurrent abortion and vulval cyst, respectively. All subjects were recruited after informed consent and none of them received any hormonal treatment for at least 3 months prior to the recruitment.

For the 30 recruited patients with endometriosis, their endometriotic lesions were harvested and fixed in neutral-buffered formalin for histologic, immunohistochemical, and histochemical analyses. In addition, 5 mL of peripheral blood samples were collected from all patients with endometriosis before and 3 months after the surgical removal of all endometriotic lesions, a time period that was long enough for full recovery. For controls, an identical amount of blood sample was obtained. All collected peripheral blood samples were processed with anticoagulant EDTA and then centrifuged at 3000 rpm for 10 minutes at room temperature to obtain plasma for analysis.

All methods and procedures employed in this study strictly adhered to the ethical principles outlined by the Helsinki Declaration and was approved by the institutional ethics review board of Shanghai OB/GYN Hospital, Fudan University.

### Immunohistochemistry analysis

The fixed human and mouse endometriotic lesion samples and mouse uterine tissues were dehydrated and embedded in paraffin, serial 4-μm sections were obtained from each block for immunohistochemistry (IHC) analysis. Routine deparaffinization and rehydration of the tissue sections were performed as reported previously (4). For antigen retrieval, the sections were heated at 98 °C in a citrate buffer (pH 6.0) for a total of 30 min for staining for HMGB1, TLR4, p-p65 and PCNA or an EDTA-Tris buffer (pH 9.0, Shanghai Sun BioTech Company, Shanghai, China) for a total of 20 min for staining for IL-33, and cooled naturally to the room temperature. For OPN staining, no antigen retrieval was needed. The tissue sections were incubated at room temperature with Trypsin Enzymatic Antigen Retrieval Solution (Ab970, Abcam) for a total of 20 min for antigen retrieval for RAGE staining. Endogenous peroxidase was blocked by incubating with 3% H_2_O_2_ for 10 min and non-specific antibody binding was blocked by incubating with 10% normal goat serum (Sunpoly-HII; BioSun Technology Co, Ltd, Shanghai, China) for 30 min at room temperature, and then incubated at 4 °C overnight with primary antibodies against HMGB1, TLR4, RAGE, p-p65, PCNA, OPN or IL-33. Sections were then washed with PBS and incubated with the horse radish peroxidase (HRP) labeled secondary antibody Detection Reagent (Sunpoly-HII) at room temperature for 30 min. The antigen-antibody complexes were stained for 3–5 min or until appropriate for microscopic examination with diaminobenzidine and then counter stained with haematoxylin (60 sec) and mounted. The information on these antibodies and their diluted concentrations used in this study, along with catalog numbers, are listed in Table 4.

**Table 4:** Information on antibodies used in this study.

Antibody name	Catalog number	Dilution	Company name
HMGB1	10829-1-AP	1:200	Proteintech
TLR4	Ab22048	1:100	Abcam
RAGE	Ab3611	1:100	Abcam
NF-κB p65 (phosphorylated)	Ab86299	1:300	Abcam
PCNA	Ab29	1:50	Abcam
OPN	Ab8448	1:300	Abcam
IL-33	Ab54385	1:100	Abcam

Images were obtained with a microscope (Olympus BX53, Olympus, Tokyo, Japan) fitted with a digital camera (Olympus DP73, Olympus). Four randomly selected images at 400X magnification of each tissue section were taken to obtain a mean optical density value by Image Pro-Plus 6.0 (Media Cybernetics, Inc., Bethesda, MD, USA), as reported previously^97^. To minimize potential bias, the person who performed IHC analysis was blinded to the group identity before analysis.

For positive controls, human breast carcinoma tissues were used for p-p65, PCNA and OPN staining, human tonsil tissues, for HMGB1 and IL-33 staining, mouse lung tissues, for RAGE staining, and mouse small intestine tissues, for TLR4 staining. For negative controls, mouse endometriotic lesion tissues were incubated with rabbit or mouse serum instead of primary antibodies. The positive and negative staining results are provided in Supplementary Fig. S2.

### Masson trichrome staining

Masson trichrome staining was used for the quantification of collagen fibers in endometriotic lesions. Tissue sections were deparaffinized in xylene and rehydrated in a graded alcohol series, then were mordant in Bouin’s solution at 37 °C for two hours, which was made with 75 mL of saturated picric acid, 25 mL of 10% formalin solution (v/v), and 5 mL of acetic acid. Sections were then stained using the Masson trichrome staining kit (Baso, Wuhan, China) according to the manufacturer’s instructions. The areas of the collagen fibers layer stained in blue relative to the entire portion of the ectopic endometrium were calculated by the Image Pro-Plus 6.0 (Media Cybernetics, Inc).

### Quantification of plasma HMGB1, OPN and HA levels

The plasma levels of HMGB1, OPN and HA in both humans and mice were quantitated using the commercial sandwich enzyme-linked immunosorbent HMGB1 (MyBioSource, San Diego, CA, USA), OPN (R&D systems), and HA (R&D systems) ELISA kits following manufacturer’s instructions. The absorbance was read at 450 nm and the reading for each standard and sample subtracted the zero standard optical density (O.D.), and then the standard curve was created by reducing the data using computer software to generate a four-parameter logistic curve-fit. Except HMGB1 concentration measured in mice, which was expressed in pg/mL, all other concentrations were expressed in ng/mL.

### Statistical analysis

The comparison of distribution of continuous variables between or among two or more groups was made using Wilcoxon’s and Kruskal’s test, and the paired Wilcoxon test, whenever appropriate, was used when the before-after comparison was made for the same group of subjects. Pearson’s or Pearson’s rank correlation coefficient was used when evaluating correlations between two variables when both variables were continuous or at least one variable was ordinal. Multiple linear regression analysis was used when multiple factors were considered. Jonckheere-Terpstra trend test was used to test the trend of more severity of dysmenorrhea. Linear discriminant analysis and multi-dimensional scaling were used to classify mice and human subjects with and without endometriosis. To determine which co-variables were associated with the severity of dysmenorrhea, we treated the severity as if it were uncensored “survival time” and used the Cox proportional hazard regression model^98^.

A p-value < 0.05 was considered statistically significant. All computations were made with R statistics software system version 3.5.1^99^ (www.r-project.org).

## Supplementary information

Supplementary figures
