# Supplementary material for: Plasma High Mobility Group Box 1 (HMGB1), Osteopontin (OPN), and Hyaluronic Acid (HA) as Admissible Biomarkers for Endometriosis
Source: Sci Rep. 2019 Jun 25;9:9272. doi: 10.1038/s41598-019-45785-w (PMC6592882; doi:10.1038/s41598-019-45785-w)
Supplement: Supplementary file 1 — Supplementary figures [file 41598_2019_45785_MOESM1_ESM.pdf]

Plasma High Mobility Group Box 1 (HMGB1), Osteopontin (OPN), and Hyaluronic Acid (HA) as Admissible Biomarkers for Endometriosis

Yunlei Cao, M.D., Xishi Liu, M.D., Ph.D., Sun-Wei Guo, Ph.D.

Supplementary Figures

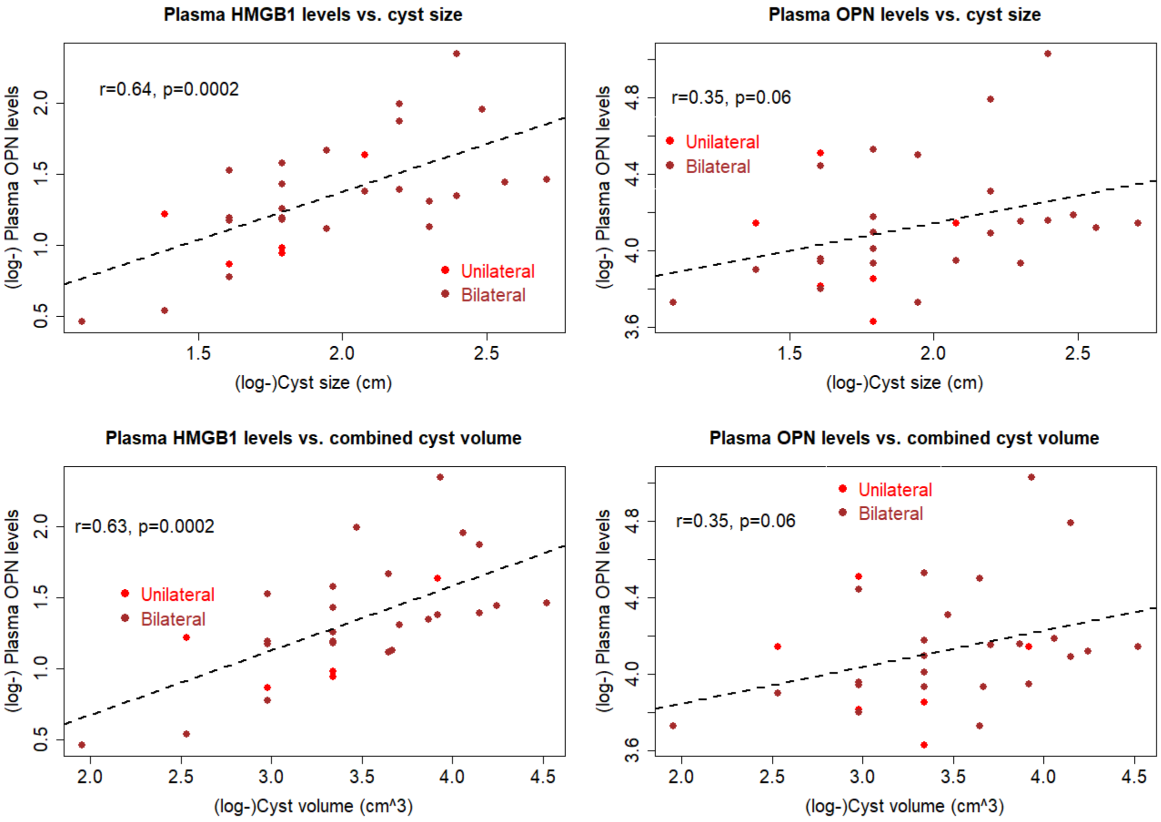

Supplementary Figure S1. Relationship between the plasma HMGB1/OPN levels and cyst sizes/volumes. Scatter plot showing the relationship between the cyst size and plasma HMGB1 (A) and OPN (B) levels, and between the combined cyst volumes and plasma HMGB1 (C) and OPN (D) levels. The dashed line represents the linear regression line, and the number is the Pearson's correlation coefficient, followed by the symbols of statistical significance levels: *: $p<0.05$; **: $p < 0.01$; ***: $p<0.001$. Each dot in the figure represents one data point/patient, with the red and brown dots indicating the patient had unilateral and bilateral ovarian endometriomas.

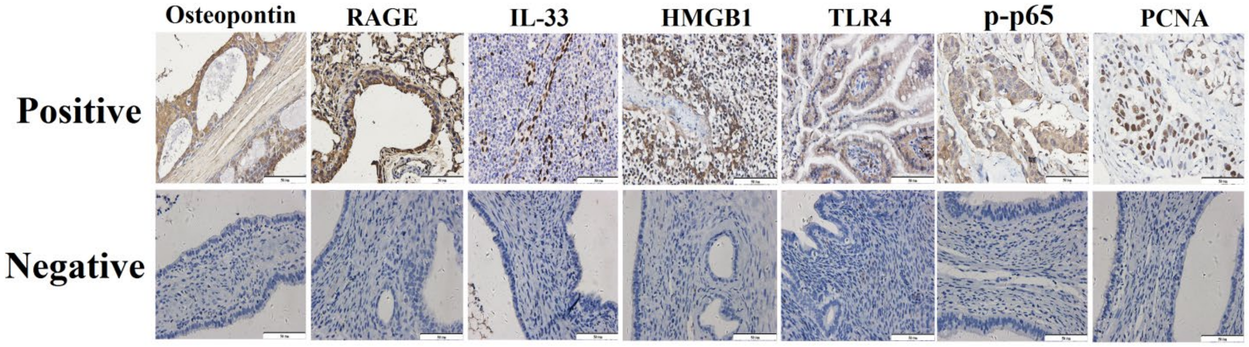

Supplementary Figure S2. Positive and negative controls for immunohistochemistry analysis. Bar=50 μm.
